# From Genetic Diagnosis to Therapeutic Implementation in Retinal Diseases: Translational Advances and Persistent Bottlenecks

**DOI:** 10.3390/biomedicines14040782

**Published:** 2026-03-30

**Authors:** Feliciana Menna, Corrado Pinelli, Laura De Luca, Alessandro Meduri, Antonio Baldascino, Stefano Lupo, Enzo Maria Vingolo

**Affiliations:** 1 Department of Medical-Surgical Sciences and Biotechnologies, U.O.C. Ophthalmology, Sapienza University of Rome, 04019 Terracina, Italy; 2 Department of Biomedical and Dental Science and of Morphological and Functional Images, University of Messina, 98122 Messina, Italy; 3 Ophthalmology Unit, Fondazione Policlinico Universitario A. Gemelli IRCCS, 00168 Rome, Italy

**Keywords:** ocular genetics, inherited retinal diseases, next-generation sequencing, epigenetics, non-coding RNA, mitochondrial dysfunction, gene therapy, genome editing, pharmacogenomics, precision ophthalmology

## Abstract

**Background**: Retinal and optic nerve disorders are a leading cause of irreversible visual impairment worldwide. Advances in molecular genetics—including next-generation sequencing, genome-wide association studies, and gene-based therapeutic technologies—have reshaped understanding of both inherited and complex retinal diseases. However, translating genetic discovery into sustained clinical benefit remains biologically and practically constrained. **Methods**: A structured literature search was conducted using PubMed and Scopus to identify relevant studies published between 2015 and 2025. The search focused on molecular genetics, epigenetic modulation, mitochondrial biology, and translational applications in inherited retinal dystrophies and selected complex retinal diseases, prioritizing high-impact original research and systematic reviews addressing diagnostic innovation and therapeutic development. **Results**: Inherited retinal dystrophies represent the most advanced model of precision ophthalmology, with diagnostic yields approaching 70–80% in well-characterized cohorts. Gene augmentation and genome-editing strategies have demonstrated proof-of-concept efficacy, yet clinical benefit depends on residual cellular viability, delivery efficiency, and durability of expression. Emerging platforms include AAV-mediated gene transfer, in vivo CRISPR-based editing, RNA-directed splice modulation, and mitochondrial-targeted approaches. Persistent barriers include unresolved non-coding and structural variants, variant interpretation uncertainty, and endpoint selection in clinical trials. In contrast, complex retinal diseases such as glaucoma, age-related macular degeneration, and pathological myopia reflect polygenic susceptibility interacting with environmental and aging-related factors. Although polygenic risk scores refine probabilistic prediction, their utility is limited by ancestry bias and incomplete predictive performance. Epigenetic and mitochondrial mechanisms further modulate disease expression but remain largely non-actionable in routine practice. **Conclusions**: Retinal genetics has progressed from gene discovery to early therapeutic implementation. Future advances will depend on improved variant detection, functional validation, biomarker-guided staging, and integration of genomics with imaging and longitudinal modeling to achieve durable and equitable precision ophthalmology.

## 1. Introduction

Visual impairment remains a major global health burden, affecting more than 2.2 billion individuals worldwide [1,2]. Inherited retinal dystrophies (IRDs) are among the leading causes of irreversible vision loss and represent a unique paradigm in medicine: a group of genetically defined diseases in which molecular diagnosis can directly enable prognostic counseling, trial eligibility, and—in selected conditions—gene-based treatment [3,4].

Over the last decade, next-generation sequencing (NGS) has revolutionized the diagnostic landscape of monogenic retinal disorders by enabling high-throughput interrogation of disease genes with broad clinical applicability [5,6]. This progress has improved molecular confirmation rates, refined genotype–phenotype correlations, and expanded eligibility for gene- and RNA-based clinical trials [7,8]. However, translation from genotype to clinical action remains uneven. A clinically meaningful fraction of patients remains genetically “unsolved,” while many “solved” cases still face uncertainty due to variant interpretation, incomplete genotype–phenotype correlations, and limited functional validation. In parallel, therapeutic pipelines have expanded rapidly (AAV gene augmentation, antisense/RNA approaches, and genome editing), but durability, immune responses, delivery constraints, and disease-stage dependency continue to limit broad clinical impact [9,10].

Accordingly, this review focuses on IRDs as a translational model, emphasizing the practical pathway from sequencing to therapy and the critical bottlenecks that determine real-world utility. This review does not aim to provide an encyclopedic catalog of all genetic eye diseases, nor to comprehensively cover all complex ocular disorders. Instead, we use selected IRD examples (retinitis pigmentosa, Leber congenital amaurosis, and Stargardt disease) to illustrate how diagnostic technologies and mechanistic insights can—and sometimes cannot—be converted into effective therapies, highlighting key knowledge gaps and controversies that define the research agenda.

The aim of this review is to analyze how genetic discoveries are directly reshaping diagnostic workflows and accelerating therapeutic pipelines in retinal and optic nerve diseases. Rather than providing an encyclopedic catalog of ocular genetics, we adopt a disease-centered and mechanism-driven perspective to highlight translationally actionable pathways, persistent implementation barriers, and the knowledge gaps that most strongly influence clinical decision-making.

## 2. Inherited Monogenic Ocular Diseases

Inherited retinal diseases (IRDs) represent the most advanced and instructive field within ocular molecular genetics, not merely because they are predominantly monogenic, but because they provide a direct and testable bridge between genetic diagnosis and therapeutic intervention. Over the past decade, IRDs have evolved from being considered rare untreatable conditions to becoming the principal proof-of-concept for precision medicine in ophthalmology. However, their translational trajectory also reveals persistent limitations that extend beyond gene discovery.

Although IRDs are genetically heterogeneous, they converge biologically on a limited number of cellular vulnerabilities: phototransduction imbalance, ciliary trafficking dysfunction, defective RNA processing, impaired outer-segment renewal, and toxic metabolite accumulation (Table 1) [7]. This convergence is clinically relevant because it suggests that disease classification should move beyond gene lists toward mechanism-based stratification, which is more informative for prognosis and therapeutic design.

At the same time, molecular resolution does not automatically equate to therapeutic readiness. Even in the era of high diagnostic yield through next-generation sequencing (NGS), a proportion of cases remain genetically unresolved due to deep-intronic, structural, or regulatory variants not captured by standard approaches [32,33,34,35]. Moreover, variant interpretation and genotype–phenotype correlations remain imperfect, particularly in disorders characterized by variable expressivity and incomplete penetrance. Thus, IRDs highlight a critical translational principle: the bottleneck has shifted from gene identification to functional interpretation and biological contextualization [36].

### 2.1. Retinitis Pigmentosa: Heterogeneity as a Translational Challenge

Retinitis pigmentosa (RP) is the paradigmatic example of how extreme genetic heterogeneity can complicate translational medicine despite extensive molecular knowledge. More than 90 genes have been implicated in RP, encompassing defects in phototransduction, ciliary transport, RNA splicing, and structural maintenance of the outer segment [4]. Yet the clinical endpoint—progressive rod–cone degeneration—appears deceptively uniform.

This convergence of phenotype masks profound mechanistic diversity. Mutations affecting phototransduction or disk morphogenesis disrupt outer-segment homeostasis, leading to metabolic overload and oxidative stress. Ciliary protein defects impair intracellular trafficking, causing mislocalization and accumulation of toxic intermediates [4]. Splicing-factor mutations selectively affect retinal cells despite ubiquitous gene expression, reflecting the retina’s exceptionally high transcriptional demand [16,17,18]. These mechanistic differences are not merely academic distinctions—they determine the plausibility, timing, and expected durability of therapeutic strategies.

A further translational obstacle in RP is the substantial proportion of genetically “unsolved” cases. Standard NGS panels may fail to detect deep-intronic variants, copy-number changes, or structural rearrangements [37,38]. Therefore, a negative molecular result does not necessarily indicate absence of genetic causation but may reflect technological or interpretative limitations. Integrating refined phenotyping with comprehensive molecular testing has been shown to improve prognostic stratification and therapeutic eligibility, as demonstrated in recent clinical cohorts [12].

Importantly, RP also exposes the limitation of traditional clinical endpoints. Visual acuity often remains preserved until advanced stages, rendering it insensitive to early rod dysfunction. This endpoint mismatch can obscure therapeutic effects and complicate trial interpretation. Thus, RP underscores the necessity of aligning molecular mechanism, disease stage, and outcome measures within a coherent translational framework [39,40].

### 2.2. Leber Congenital Amaurosis: Timing and Reversibility

Leber congenital amaurosis (LCA) represents the most severe and earliest-onset form of inherited retinal degeneration [13,14]. Pathogenic variants in genes such as CEP290, GUCY2D, CRB1, RDH12, and RPE65 disrupt essential processes including phototransduction recovery, retinoid cycling, and ciliary integrity [11,26,27,41,42,43].

The principal translational insight from LCA lies in distinguishing between functional impairment and structural loss. In RPE65-associated disease, dysfunction of the visual cycle can be partially reversed when photoreceptors remain viable, as demonstrated by the clinical success of voretigene neparvovec [12]. This example confirmed that targeted gene augmentation can yield meaningful functional improvement when delivered within an appropriate therapeutic window.

However, not all LCA subtypes are equally amenable to such intervention. Conditions characterized by early structural disruption may not benefit from late-stage molecular correction, even when the causative variant is known. This reality highlights that genetic diagnosis must be interpreted alongside biomarkers of residual cellular integrity. Furthermore, genome-editing approaches targeting CEP290-associated LCA have demonstrated feasibility but continue to face challenges related to editing efficiency, immune response, and long-term durability. Thus, LCA illustrates both the promise and the biological constraints of precision gene-based therapy.

### 2.3. Stargardt Disease: Toxic Metabolite Accumulation and Functional Stratification

Stargardt disease (STGD1), caused by biallelic ABCA4 variants, exemplifies a toxicity-driven model of retinal degeneration [3,19]. Impaired ABCA4 function results in accumulation of bisretinoid by-products such as A2E within retinal pigment epithelium (RPE), promoting lipofuscin deposition and progressive macular atrophy [20,21,22,23,32].

Unlike disorders driven purely by loss-of-function, STGD1 severity depends heavily on the biochemical impact of individual alleles rather than simply their presence. High allelic heterogeneity complicates variant interpretation and generates a wide phenotypic spectrum. Consequently, functional classification of variants becomes essential for prognostic counseling and trial stratification.

STGD1 also illustrates the importance of imaging biomarkers in translational research. Fundus autofluorescence enables noninvasive quantification of lipofuscin distribution and atrophic progression, providing objective outcome measures aligned with disease biology [44,45]. Therapeutic strategies under investigation—including RNA-based approaches and modulation of the visual cycle—reflect the need to address toxic accumulation rather than merely restore gene expression.

Thus, STGD1 reinforces a broader translational principle: therapeutic design must follow disease mechanism, and not all monogenic diseases are optimally treated by straightforward gene augmentation.

### 2.4. Next-Generation Sequencing: Beyond Diagnostic Yield

NGS has profoundly transformed the diagnostic approach to IRDs by enabling simultaneous interrogation of numerous disease-associated genes [32,33,34]. Targeted panels typically achieve diagnostic yields between 50% and 70%, while whole-exome sequencing (WES) may approach 80% in carefully phenotyped cohorts [35,36]. Whole-genome sequencing (WGS) can further identify deep-intronic, structural, and copy-number variants undetectable by other methods.

However, increasing diagnostic yield is only one dimension of clinical impact. Variant interpretation remains a substantial bottleneck, particularly in the context of variants of uncertain significance (VUS) and non-coding alterations. RNA-based analyses can uncover splicing defects not predicted by DNA sequencing alone, but such approaches are not yet standardized in routine practice.

Therefore, the current challenge in IRD genetics is less about sequencing capacity and more about integrating molecular findings with phenotypic data, longitudinal imaging, and functional validation. Without such integration, even comprehensive genomic data may fail to translate into actionable clinical decisions. The principal diagnostic strategies currently used in inherited and complex ocular diseases are summarized in Table 2.

## 3. Complex and Polygenic Eye Diseases

Complex ocular diseases such as glaucoma, age-related macular degeneration (AMD), and pathological myopia differ fundamentally from inherited retinal dystrophies in their genetic architecture and translational trajectory [46,47,48,49,50]. Whereas IRDs typically arise from high-penetrance variants in single genes, complex retinal disorders reflect the cumulative effect of numerous common variants of modest effect interacting with environmental exposure, aging-related processes, and systemic comorbidities. Genome-wide association studies (GWAS) have reshaped the conceptual framework of these diseases by identifying hundreds of risk loci and clarifying biological pathways involved in inflammation, extracellular matrix remodeling, mitochondrial resilience, vascular regulation, and neuronal survival [5,9,51,52,53,54,55,56,57,58,59]. However, the clinical translation of these findings remains limited, not because of insufficient genetic discovery, but because the relationship between genotype and phenotype is probabilistic rather than deterministic.

### 3.1. Glaucoma: Genetic Risk as a Modifier of Vulnerability

Glaucoma exemplifies the complex interplay between genetic predisposition and mechanical stress. Elevated intraocular pressure (IOP) remains the principal modifiable risk factor, yet individuals with comparable IOP levels may experience markedly different disease trajectories. This variability reflects underlying genetic susceptibility that influences trabecular meshwork function, optic nerve head biomechanics, mitochondrial function, and retinal ganglion cell (RGC) resilience [5,6,9].

Rare monogenic variants, such as those affecting MYOC or OPTN, have provided mechanistic insight into endoplasmic reticulum stress, impaired protein folding, defective autophagy, and enhanced RGC vulnerability to oxidative injury [5,6,60,61]. These discoveries clarified pathogenic pathways but account for only a small fraction of overall disease burden. The majority of primary open-angle glaucoma is polygenic, with GWAS identifying numerous loci that collectively modulate IOP regulation and optic nerve susceptibility [9]. Importantly, the effect size of individual variants is modest, and their predictive capacity emerges only when aggregated into polygenic risk scores (PRSs).

Although polygenic risk scores are increasingly proposed for risk stratification in glaucoma, their clinical implementation remains constrained by limited transferability across ancestries. Most discovery datasets remain enriched for individuals of European ancestry, and PRS performance can decline substantially when applied to underrepresented populations, thereby risking systematic misclassification and potential amplification of health disparities. This limitation has both ethical and practical implications: adopting PRS-driven surveillance strategies without robust multi-ancestry calibration could preferentially benefit populations already well represented in genomic resources. Consequently, the most defensible near-term role of PRSs in glaucoma is as an adjunct to structural and functional phenotyping within ancestry-aware models, rather than as a stand-alone tool for individualized clinical decision-making [62,63,64,65]. Furthermore, performance varies significantly across ancestries, reflecting the demographic composition of discovery cohorts. Thus, while genomic profiling refines risk stratification, glaucoma management remains primarily phenotype-driven. The principal translational challenge lies in integrating genetic risk with structural imaging and functional testing to identify patients in whom neuroprotective strategies may be particularly relevant.

### 3.2. Age-Related Macular Degeneration: Complement Biology and Incomplete Therapeutic Alignment

AMD represents the most extensively studied complex retinal disease from a genetic perspective. Variants in complement-related genes—most prominently CFH Y402H—and in the ARMS2/HTRA1 locus account for a substantial proportion of heritable susceptibility [8,9,10,66,67,68,69,70,71,72]. These discoveries established chronic complement dysregulation and extracellular matrix remodeling as central biological axes of disease pathogenesis. Experimental evidence indicates that altered complement regulation at the retinal pigment epithelium (RPE) interface promotes sustained low-grade inflammation, drusen formation, and progressive RPE dysfunction [44].

Despite these advances, therapeutic translation has not fully aligned with genetic insight. Anti-VEGF therapy has transformed the management of neovascular AMD, primarily by targeting downstream angiogenic processes. However, upstream mechanisms such as complement dysregulation and inflammation play a central role in disease pathobiology [73,74]. Complement inhibitors are under active investigation, yet patient selection remains challenging because genetic risk does not linearly predict disease progression or treatment response [75,76,77]. Moreover, AMD progression reflects cumulative oxidative stress, mitochondrial dysfunction, and environmental exposures such as smoking and dietary factors [78,79], which are only partially captured by genetic profiling.

Similarly, while genetic risk modeling and PRSs can improve probabilistic prediction of AMD progression, generalizability remains a central barrier. The majority of AMD PRSs have been developed in predominantly European cohorts, and reduced predictive performance in other ancestries raises concerns regarding equity and clinical validity when deployed broadly. In AMD, where environmental exposure and aging-related processes strongly shape disease trajectory, clinically meaningful prediction is most likely to emerge from integrated frameworks that combine genotype with imaging biomarkers and modifiable risk factors, rather than from genomic scores alone [80,81].

This gap between genetic discovery and therapeutic implementation highlights a broader issue: identifying risk loci does not automatically identify actionable targets. The clinical utility of genomic data in AMD may therefore lie more in risk prediction models that integrate genotype with imaging biomarkers and systemic risk factors, rather than in single-pathway intervention strategies [82,83].

### 3.3. Pathological Myopia: Polygenicity in the Context of Environmental Amplification

Pathological myopia illustrates the limits of genetic determinism in the presence of strong environmental modulation [15,79]. Large-scale GWAS have identified hundreds of loci associated with refractive error and axial length, implicating extracellular matrix organization, neuronal development, and light-induced signaling pathways in ocular growth regulation [51,52,53,54,55,56,57,58,59,84,85]. These findings confirm the highly polygenic nature of refractive error and underscore the biological complexity of scleral remodeling.

However, the dramatic global rise in myopia prevalence cannot be attributed solely to genetic predisposition. Environmental factors—particularly near-work intensity and reduced outdoor light exposure—interact with genetic susceptibility to influence axial elongation. Epigenetic studies have reported DNA methylation alterations in genes associated with metabolic regulation and oxidative stress in high myopia [86,87], while experimental models implicate hypoxia and dysregulated TGF-β signaling in scleral fibroblast activation and biomechanical remodeling [88,89].

Thus, in pathological myopia, genetics defines a susceptibility framework rather than a deterministic outcome. Preventive strategies remain largely behavioral and environmental, even as genomic data refine understanding of underlying biology. The translational frontier lies in identifying individuals at highest risk of excessive elongation early enough to implement targeted interventions, rather than in gene-specific therapeutic correction.

### 3.4. Polygenic Risk Scores: Promise and Structural Limitations

The development of polygenic risk scores represents a logical extension of GWAS discoveries. By aggregating the cumulative effect of numerous small-effect variants, PRSs aim to quantify individual genetic susceptibility to complex diseases [62,63,64,65,90,91,92,93]. In glaucoma, PRSs can identify individuals at elevated genetic risk even in the absence of elevated IOP, potentially supporting intensified surveillance strategies [62,63,64,65]. In AMD, PRSs correlate with progression to advanced stages and may complement imaging-based grading systems [90,91]. Similar predictive approaches have been explored in myopia to identify children at increased risk of high axial elongation [92,93].

Nevertheless, several structural limitations restrict immediate clinical adoption. Predictive performance declines outside the ancestral populations in which scores were derived, limiting equity and generalizability. PRSs do not provide diagnostic certainty and cannot replace phenotypic evaluation. Furthermore, ethical considerations arise regarding genetic risk disclosure in diseases for which preventive options may be limited or variably effective.

Consequently, the most realistic near-term application of PRSs in ophthalmology is as an adjunctive stratification tool within integrated predictive models that incorporate imaging, environmental exposure, and systemic health data. Complex retinal diseases therefore exemplify a translational paradigm distinct from monogenic IRDs: genetic architecture informs biological understanding and probabilistic risk, but therapeutic implementation requires multi-dimensional integration beyond genomics alone.

## 4. Epigenetic Regulation in Retinal and Optic Nerve Disorders: Modulation, Plasticity, and Translational Uncertainty

Epigenetic regulation has emerged as an important layer of biological modulation in retinal and optic nerve diseases, particularly in conditions characterized by variable penetrance, age dependency, or strong environmental influence. Unlike germline sequence variants, epigenetic modifications—including DNA methylation, histone modification, and chromatin remodeling—do not alter nucleotide sequence but influence gene expression dynamics in response to metabolic, inflammatory, and environmental stimuli [94,95]. In the context of retinal disease, the critical question is not whether epigenetic alterations occur—they clearly do—but whether they function as primary pathogenic drivers or secondary modifiers of genetically determined vulnerability.

In age-related macular degeneration (AMD), altered methylation profiles have been reported in complement pathway genes, including CFH and C3, reinforcing the established genetic link between complement dysregulation and chronic retinal inflammation [96,97,98]. However, most studies demonstrate association rather than causation. It remains unclear whether methylation changes initiate inflammatory cascades or reflect downstream adaptation to oxidative stress and cellular injury. This distinction is not merely academic; therapeutic strategies targeting epigenetic pathways require demonstration of upstream regulatory control rather than reactive epiphenomena.

Similarly, in pathological myopia, epigenetic modulation of TGF-β–related signaling pathways has been implicated in scleral remodeling and axial elongation [58]. Experimental models suggest that environmental stressors, including altered visual input and hypoxia, may induce epigenetic shifts that modify extracellular matrix composition and biomechanical properties [86,87,88,89]. These findings offer a plausible mechanistic bridge between genetic predisposition and environmental amplification. Yet longitudinal human data demonstrating stable, disease-driving epigenetic signatures remain limited, and inter-individual variability complicates reproducibility.

In glaucoma, emerging evidence suggests that epigenetic mechanisms may modulate retinal ganglion cell vulnerability under conditions of oxidative stress and mechanical strain [99]. Altered chromatin states and histone modifications have been linked to stress-response gene expression, but the extent to which these changes precede neurodegeneration rather than follow it remains uncertain. As in AMD and myopia, the directionality of epigenetic influence is difficult to establish in human tissue, given that most data derive from advanced disease stages.

Non-coding RNAs (ncRNAs) add another regulatory layer influencing post-transcriptional control of inflammation, angiogenesis, and extracellular matrix turnover. MicroRNAs such as miR-21, miR-29b, miR-146a, and miR-204 have been implicated in AMD-related inflammatory signaling and trabecular meshwork homeostasis in glaucoma [100,101,102,103,104,105]. Long non-coding RNAs and circular RNAs have been associated with retinal vascular remodeling and oxidative stress responses in experimental models [106,107]. While these observations support biological plausibility, functional validation in human retinal tissue remains limited. In many cases, ncRNA dysregulation may amplify pre-existing pathogenic processes rather than initiate them.

From a translational perspective, epigenetic and ncRNA research offers both opportunity and caution. On one hand, epigenetic modulation is theoretically reversible, rendering it an attractive therapeutic target. Histone deacetylase inhibitors and RNA-based therapeutics have demonstrated neuroprotective effects in preclinical models [108,109,110,111,112,113]. On the other hand, systemic epigenetic manipulation carries risks of off-target effects, and tissue-specific delivery remains challenging. Moreover, because epigenetic states are dynamic and context-dependent, therapeutic timing and patient selection become critical variables [114,115,116,117].

The most defensible interpretation of current evidence is that epigenetic and non-coding RNA alterations primarily function as modifiers of disease expression and progression rather than as independent primary causes. They help explain incomplete penetrance, variable severity, and environmental susceptibility across retinal diseases, but they do not replace the foundational role of genomic variation in initiating pathology. Consequently, while epigenetic therapies hold conceptual promise, their translation into clinical ophthalmology remains exploratory and largely preclinical. Beyond mechanistic interpretation, an increasingly relevant translational direction is the use of epigenetic signatures as candidate biomarkers for early detection or risk stratification. Because DNA methylation states can reflect cumulative exposure to oxidative stress, inflammation, and metabolic perturbations, epigenetic profiles may capture aspects of disease vulnerability not fully explained by germline variation alone [94,95]. However, biomarker utility will depend on longitudinal validation, tissue and cell-type specificity, and reproducibility across cohorts, as epigenetic patterns measured in accessible tissues may not faithfully mirror retinal states. Thus, while epigenetic biomarkers are a promising avenue for earlier risk assessment, current evidence remains insufficient for routine clinical implementation and should be interpreted cautiously as predominantly correlative [96,97,98,99,109,118,119].

In this sense, epigenetics should be viewed not as a parallel pathogenic axis competing with genomics, but as an integrative regulatory layer that modulates the phenotypic expression of both monogenic and polygenic retinal disease.

## 5. Mitochondrial Genetics and Optic Neuropathies: Energetic Vulnerability, Incomplete Penetrance, and Therapeutic Frontiers

Hereditary optic neuropathies provide a biologically distinct yet conceptually aligned model within ocular genetics, in which mitochondrial integrity represents the central determinant of neuronal survival. Retinal ganglion cells (RGCs) are uniquely vulnerable to bioenergetic stress due to their high metabolic demand, long unmyelinated intraocular axons, and dependence on tightly regulated oxidative phosphorylation. Consequently, even subtle impairments in mitochondrial function can precipitate selective RGC degeneration [11,24,25].

Unlike many inherited retinal dystrophies driven by structural photoreceptor defects, mitochondrial optic neuropathies highlight the role of cellular energy failure and reactive oxygen species (ROS) imbalance as primary pathogenic triggers. This distinction has profound translational implications: restoring structural proteins may not suffice if bioenergetic resilience is not simultaneously addressed.

### 5.1. Leber Hereditary Optic Neuropathy: Mutation Is Necessary but Not Sufficient

Leber hereditary optic neuropathy (LHON) is classically caused by point mutations in mitochondrial DNA (mtDNA) affecting complex I subunits—most frequently MT-ND1, MT-ND4, and MT-ND6—resulting in impaired oxidative phosphorylation and excess ROS production [25,26,27]. However, LHON challenges simplistic genotype–phenotype paradigms because mutation carriage does not equate to disease manifestation. Incomplete penetrance and marked male predominance underscore the modifying influence of nuclear genetic background, hormonal factors, and environmental triggers such as smoking and alcohol exposure.

This incomplete penetrance is not a peripheral observation; it is central to understanding mitochondrial disease biology. The pathogenic mtDNA mutation creates a state of energetic vulnerability, but disease onset appears to require additional stressors that exceed compensatory capacity. From a translational perspective, this suggests that therapeutic strategies must not only correct the primary mutation but also enhance mitochondrial resilience and antioxidant defenses.

Idebenone, a synthetic quinone approved in Europe for LHON, partially bypasses complex I dysfunction by facilitating electron transfer within the respiratory chain [42]. Clinical benefit is most pronounced when administered early, reinforcing the principle that therapeutic timing is critical. However, response variability remains substantial, reflecting heteroplasmy levels, disease stage, and nuclear modifiers.

Gene therapy targeting MT-ND4 through allotopic expression delivered by AAV2 vectors has demonstrated bilateral functional improvement despite unilateral injection, possibly due to vector diffusion or transsynaptic spread [43]. While these findings represent a milestone in mitochondrial-targeted therapy, important questions persist regarding long-term expression stability, mitochondrial import efficiency, and the durability of functional gains. Thus, LHON illustrates both the feasibility and the biological complexity of targeting mitochondrial genomes.

### 5.2. Dominant Optic Atrophy: Mitochondrial Dynamics and Structural Fragility

Dominant optic atrophy (DOA), most commonly caused by mutations in OPA1, provides a complementary perspective on mitochondrial pathology [28,120,121]. OPA1 is a key regulator of mitochondrial inner-membrane fusion, cristae architecture, and apoptosis control. Loss of functional OPA1 leads to mitochondrial fragmentation, impaired ATP synthesis, and increased susceptibility to apoptotic signaling within RGCs [29].

Unlike LHON, which primarily disrupts electron transport, DOA emphasizes the importance of mitochondrial network dynamics and structural integrity. The phenotypic spectrum ranges from isolated optic atrophy to multisystemic “DOA-plus” syndromes, reflecting systemic mitochondrial vulnerability [29]. Experimental restoration of OPA1 expression has been shown to rescue mitochondrial morphology and improve neuronal survival in preclinical models [30]. However, translating these findings into human therapy requires overcoming challenges related to efficient gene delivery to RGCs and ensuring sustained expression without adverse immune responses.

The broader translational lesson from DOA is that mitochondrial dysfunction is not solely a defect in ATP production; it encompasses altered fission–fusion balance, disrupted cristae organization, and dysregulated apoptotic thresholds. Therapeutic strategies therefore need to address mitochondrial quality control rather than focusing exclusively on electron transport correction.

### 5.3. Emerging Mitochondrial Therapeutic Strategies: Promise and Biological Constraints

Beyond conventional gene augmentation, several innovative strategies aim to directly or indirectly restore mitochondrial function. Allotopic expression remains the most clinically advanced approach in LHON [43], yet challenges in efficient protein import into mitochondria and sustained expression highlight the complexity of manipulating organelle-specific genetics.

Mitochondrial genome editing represents a conceptual breakthrough, aiming to selectively modify pathogenic mtDNA variants [27]. However, technical barriers—including delivery of editing machinery into mitochondria and ensuring selective targeting without off-target cleavage—remain substantial. Similarly, approaches involving RNA import technologies seek to compensate for defective mitochondrial transcripts but are still in early experimental stages [122].

Another emerging concept is intercellular mitochondrial transfer, whereby healthy mitochondria are delivered to compromised neurons [123]. Preclinical data suggest potential neuroprotective effects, but clinical translation faces logistical and regulatory challenges, including ensuring functional integration and long-term stability.

Collectively, these strategies converge on a central theme: enhancing bioenergetic resilience may be as important as correcting primary genetic defects. In this regard, mitochondrial-targeted antioxidants, metabolic modulators, and neurotrophic support remain relevant adjunctive approaches [31,124]. Importantly, mitochondrial optic neuropathies reinforce the idea that neuroprotection and genetic correction should not be viewed as competing strategies but as complementary components of a comprehensive therapeutic framework.

### 5.4. Translational Challenges Unique to Mitochondrial Genetics

Mitochondrial diseases introduce additional complexities absent in nuclear monogenic disorders. Heteroplasmy levels influence disease severity and therapeutic response, yet accurate quantification and prediction of heteroplasmic shifts over time remain challenging. Furthermore, mtDNA is maternally inherited and lacks recombination, limiting opportunities for natural correction.

Immune considerations are also relevant, as mitochondrial proteins expressed through allotopic strategies may trigger unexpected immune responses. Long-term follow-up is therefore essential to establish safety and durability. Finally, because RGC loss is often rapid and irreversible once initiated, early detection and intervention are paramount, underscoring the need for sensitive biomarkers of subclinical mitochondrial dysfunction.

Mitochondrial optic neuropathies expand the conceptual scope of ocular genetics by shifting attention from structural protein defects to energetic vulnerability and cellular stress thresholds. They demonstrate that pathogenic mutations may create a predisposition rather than an immediate phenotype, and that disease expression often depends on cumulative metabolic burden [111].

From a translational standpoint, the future of mitochondrial therapy likely lies in combinatorial strategies that integrate genetic correction, metabolic support, and neuroprotection. As with inherited retinal dystrophies, success will depend not only on molecular precision but also on timing, delivery efficiency, and sustained functional rescue.

## 6. Gene Therapy in Retinal Diseases: Biological Alignment, Delivery Constraints, and Durability Challenges

Gene therapy has become the most visible translational outcome of retinal molecular genetics. The retina is often described as an ideal target for gene-based intervention due to its compartmentalized anatomy, relative immune privilege, and accessibility through subretinal or intravitreal delivery. However, while these anatomical features facilitate localized treatment, therapeutic success is fundamentally determined by biological alignment between disease mechanism, target cell viability, and timing of intervention rather than by vector technology alone.

Adeno-associated virus (AAV) vectors remain the dominant platform for retinal gene delivery because of their favorable safety profile and efficient transduction of photoreceptors, retinal pigment epithelium (RPE), and retinal ganglion cells [125,126]. The clinical approval of voretigene neparvovec for RPE65-associated retinal dystrophy provided proof-of-concept that gene augmentation can restore visual function when sufficient photoreceptor integrity is preserved [127,128]. Importantly, this success was not merely a triumph of vector engineering; it reflected a disease model in which functional impairment preceded irreversible structural degeneration. The therapeutic window was therefore biologically permissive.

This principle—that reversibility depends on residual cellular viability—remains central to interpreting subsequent clinical trials. In choroideremia and X-linked retinoschisis, early-phase studies demonstrated anatomical or biological effects, yet functional outcomes have been variable and, in some cases, modest [75,76]. These discrepancies underscore that successful transduction does not guarantee meaningful clinical recovery, particularly when degeneration is advanced or when the treated retinal area represents only a fraction of total affected tissue. Moreover, dose-dependent inflammatory responses observed in some programs reveal that immune privilege is relative rather than absolute, especially when higher vector loads are required to achieve therapeutic thresholds.

Achromatopsia trials targeting CNGA3 or CNGB3 further illustrate the importance of developmental timing. In congenital cone dysfunction, long-standing sensory deprivation may limit functional recovery even after gene delivery. This suggests that early intervention—potentially during developmental windows—may be necessary to maximize benefit. Thus, gene therapy outcomes are shaped not only by molecular correction but also by the neurodevelopmental history of the visual system.

Genome editing has extended the therapeutic paradigm beyond gene augmentation, enabling mutation-specific correction within the native genomic context. CRISPR-based approaches, including the EDIT-101 program for CEP290-associated LCA10, demonstrated the feasibility of in vivo retinal genome editing [27,123]. However, feasibility does not equate to predictable clinical efficacy. Editing efficiency within target cells remains variable, off-target effects require rigorous surveillance, and long-term genomic stability has yet to be fully established. Furthermore, editing strategies must balance therapeutic benefit against the risks associated with permanent genomic alteration.

An additional complexity arises from gene size constraints. AAV vectors have limited packaging capacity, rendering large genes such as ABCA4 and MYO7A challenging to deliver efficiently. Dual-vector strategies have been explored, but recombination efficiency and consistent expression remain concerns [75,76]. These technical constraints emphasize that vector choice and genomic architecture directly influence therapeutic feasibility.

Beyond monogenic IRDs, gene-based strategies are being investigated for neovascular retinal diseases through sustained intraocular expression of anti-VEGF molecules. While early-phase studies suggest reduced injection burden, sustained pathway inhibition raises concerns regarding long-term vascular homeostasis and the inability to titrate expression dynamically. In contrast to periodic intravitreal injections, gene-based anti-VEGF expression introduces a semi-permanent modulation of angiogenic signaling, the long-term implications of which remain under evaluation.

Durability represents another unresolved dimension. While short- and medium-term outcomes in certain trials are encouraging, gradual decline in functional gains has been reported in some patients treated with gene augmentation [75,76,128]. Potential explanations include ongoing degenerative processes independent of the corrected pathway, immune-mediated attenuation of expression, or insufficient panretinal coverage. These observations challenge the assumption that correcting a primary mutation necessarily halts downstream degeneration.

Importantly, gene therapy should not be conceptualized as a standalone solution but as one component within a broader therapeutic ecosystem. In advanced retinal degeneration, where photoreceptor loss is extensive, optogenetic approaches aim to confer photosensitivity to inner retinal neurons, effectively bypassing lost cells. Early clinical data suggest partial restoration of light perception, but spatial resolution and functional acuity remain limited. Such approaches shift therapeutic expectations from restoration to substitution and highlight the need to recalibrate outcome measures accordingly.

Collectively, current evidence indicates that gene therapy success depends on precise alignment among molecular mechanism, disease stage, vector capability, and endpoint selection. The field has transitioned from questioning whether gene therapy is possible to interrogating under which biological conditions it is sustainable and clinically meaningful. Future progress will likely require combinatorial strategies that integrate gene correction with neuroprotection, metabolic support, and inflammation modulation [129,130]. In addition to biological constraints, real-world implementation of retinal gene therapy is increasingly shaped by manufacturing scalability and supply-chain limitations. The production of clinical-grade AAV vectors requires complex upstream and downstream processes, stringent quality control, and substantial capacity, and scaling these workflows to meet broader clinical demand remains challenging. Batch-to-batch variability, high production costs, and regulatory requirements can limit availability and delay treatment, particularly as indications expand beyond ultra-rare diseases. These constraints represent a practical barrier to equitable access and should be considered alongside efficacy and safety when evaluating the long-term feasibility of AAV-based therapies.

Thus, retinal gene therapy has entered a maturation phase. The conceptual breakthrough has already occurred; the next challenge lies in optimizing durability, refining patient selection, and developing biomarkers capable of predicting which individuals are most likely to derive sustained benefit. Only through such biologically informed refinement will gene-based interventions achieve consistent and scalable clinical impact. Approved and investigational gene-based therapies in retinal and optic nerve diseases are summarized in Table 3.

## 7. Pharmacogenomics and Precision Therapeutics in Ophthalmology: Between Biological Plausibility and Clinical Implementation

Pharmacogenomics (PGx) represents a natural extension of molecular ophthalmology, seeking to understand how inherited genetic variation influences therapeutic response, adverse-effect susceptibility, and treatment durability. In principle, retinal diseases—particularly those requiring chronic intravitreal therapy or systemic pharmacological intervention—provide an ideal setting for precision drug stratification. In practice, however, pharmacogenomics in ophthalmology remains at an intermediate stage between biological plausibility and routine clinical implementation.

The variability in response to anti-VEGF therapy for neovascular age-related macular degeneration (nAMD) and diabetic macular edema has long suggested an underlying genetic component. Polymorphisms in VEGFA and its receptor KDR have been associated with differential anatomical and functional response in several cohorts [37,63,118,131]. Similarly, variants in inflammatory mediators and complement pathway genes—including CFH and ARMS2/HTRA1—have been explored as modulators of therapeutic effect [131,132]. Yet replication across populations has been inconsistent, and effect sizes remain modest. The complexity of angiogenic signaling, combined with environmental influences and disease-stage variability, likely attenuates the predictive power of single polymorphisms [133,134].

The introduction of faricimab, which simultaneously targets VEGF-A and Angiopoietin-2, further complicates the pharmacogenomic landscape. Theoretical models suggest that variation in ANG2-regulatory pathways could influence responsiveness to dual inhibition [80,135,136]. However, current evidence does not yet support genotype-driven therapeutic selection. Thus, while genetic variability may contribute to treatment heterogeneity, it has not reached the threshold required for standardized decision-making.

Pharmacogenomics also holds potential for anticipating adverse drug reactions, particularly in scenarios where ocular toxicity or pressure elevation can result in irreversible damage. Fingolimod-associated macular edema (FAME) provides a relevant example, as genetic variants in sphingolipid metabolism and vascular permeability pathways have been implicated in individual susceptibility [133,137]. Likewise, steroid-induced ocular hypertension has been linked to variants in MYOC and FKBP5, which modulate trabecular meshwork response to glucocorticoids [134]. These associations offer mechanistic insight into why certain individuals exhibit pronounced intraocular pressure elevation while others do not.

However, translating such findings into routine screening raises practical and ethical considerations. The predictive accuracy of these variants is not absolute, and widespread genotyping prior to therapy may not be cost-effective or clinically justified in the absence of strong prospective validation. Furthermore, risk prediction must be balanced against the availability of alternative therapies and the feasibility of enhanced monitoring.

Hydroxychloroquine-associated retinopathy illustrates a similar tension. Variants in genes involved in retinoid metabolism, such as ABCA4 and RDH8, have been proposed as potential modifiers of toxicity risk [129]. Yet large-scale prospective studies confirming actionable predictive value are lacking. Consequently, current screening guidelines rely primarily on dose, duration, and imaging surveillance rather than genetic testing.

Beyond single-gene associations, the integration of pharmacogenomics with multi-omics profiling and machine learning represents an emerging frontier [138]. Predictive models combining genotype, imaging biomarkers, and longitudinal treatment data have demonstrated improved capacity to anticipate therapeutic response in retinal vascular disease [39]. These approaches shift the paradigm from isolated polymorphism analysis toward systems-level prediction. However, model generalizability and reproducibility across institutions remain challenges, particularly when training datasets lack ethnic diversity.

An additional conceptual issue lies in distinguishing correlation from causation. Genetic associations with drug response may reflect linkage disequilibrium with nearby regulatory elements rather than direct functional modulation of pharmacodynamics. Without mechanistic validation, implementation risks overinterpreting statistical associations.

From a translational standpoint, pharmacogenomics in ophthalmology currently functions more as a hypothesis-generating framework than as a standardized clinical tool. The strongest near-term utility may lie in identifying subgroups at higher risk of adverse effects or in refining injection intervals through integrative predictive modeling rather than in dictating first-line therapy selection.

Ultimately, precision therapeutics in ophthalmology will likely depend on the convergence of pharmacogenomics, advanced imaging analytics, and longitudinal real-world evidence. Genetic variation undoubtedly contributes to interindividual variability in treatment response, but its clinical leverage will depend on rigorous validation, ancestry-diverse datasets, and integration into multidimensional decision-support systems. Until then, pharmacogenomics remains a promising yet incompletely realized component of precision ophthalmology. Key pharmacogenomic associations and their current level of clinical actionability are summarized in Table 4.

## 8. Future Perspectives

The next decade is likely to witness substantial evolution in the application of molecular genetics to ophthalmic disease, driven by the convergence of high-throughput multi-omics technologies, machine learning, and advanced genomic engineering. Integration of genomic, transcriptomic, proteomic, and metabolomic datasets will enable reconstruction of disease-specific molecular networks, allowing for earlier and more accurate identification of mechanistic bottlenecks and therapeutic targets. As these datasets expand, predictive models will increasingly incorporate longitudinal imaging and functional data, thereby enhancing clinical trial design and enabling earlier intervention in patients at highest risk of rapid progression [139,140].

Therapeutically, gene augmentation is expected to be complemented by, rather than replaced by, genome-editing approaches, as tools such as base editing and prime editing become safer and more efficient [27]. These technologies may allow correction of mutations previously considered inaccessible to AAV-based delivery and may extend the therapeutic window by targeting residual viable cells. Parallel progress in RNA-based therapies and nanoparticle delivery systems may broaden the applicability of post-transcriptional modulation to both inherited and multifactorial retinal diseases. In addition, strategies that focus on metabolic and mitochondrial support, including mitochondrial genome repair, intercellular mitochondrial transfer, and modulation of oxidative-stress pathways, are likely to become increasingly relevant in optic neuropathies and degenerative retinal disorders [41].

Artificial intelligence will play a pivotal role in integrating these complex datasets to support diagnostic interpretation, variant prioritization, and individualized therapeutic planning. Its capacity to merge genotypic, phenotypic, and imaging data will facilitate more accurate prognostic modeling and guide the timing and selection of therapies [141].

**Figure 1:**
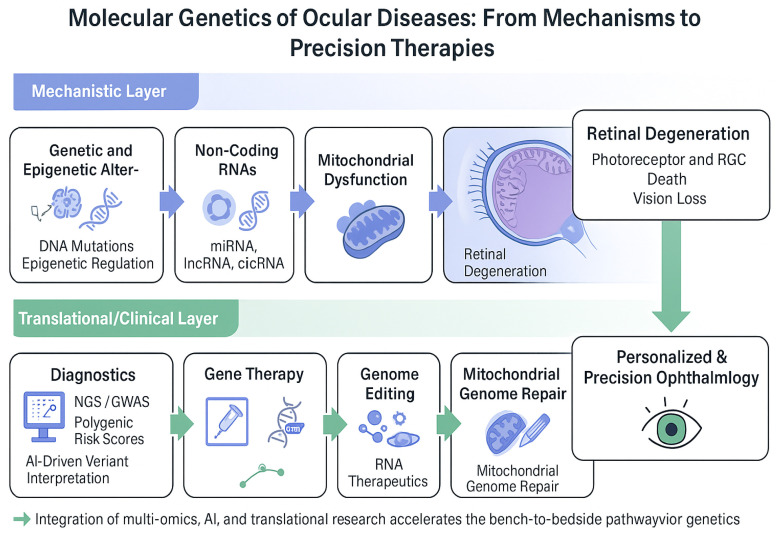
Graphical abstract illustrating the molecular-to-clinical continuum in ocular genetics. The upper “Mechanistic Layer” depicts the main pathogenic mechanisms—genetic and epigenetic alterations, non-coding RNA regulation, and mitochondrial dysfunction—leading to retinal degeneration and vision loss. The lower “Translational/Clinical Layer” highlights emerging diagnostic and therapeutic strategies, including next-generation sequencing, genome-wide association studies, gene therapy, CRISPR-based genome editing, RNA therapeutics, and mitochondrial genome repair. Integration of multi-omics technologies, artificial intelligence, and translational research drives the development of personalized and precision ophthalmology.

Ultimately, the successful translation of these innovations will depend on collaboration across disciplines, harmonization of data-sharing frameworks, and continued investment in clinical research infrastructure. As these elements coalesce, the field of ocular genetics is poised to shift from descriptive analysis to predictive and personalized intervention, redefining care pathways for inherited and complex eye diseases (Figure 1).

## 9. Conclusions

This review does not aim to catalog all genetic mechanisms implicated in ocular disease, but rather to highlight how retinal disorders have emerged as a unifying model for translating molecular genetics into clinical practice. By focusing on retinal and optic nerve diseases, we demonstrate how advances in sequencing technologies, functional genomics, and targeted therapies have reshaped diagnosis and treatment in a way not yet paralleled in other ocular subspecialties.

Molecular genetics has profoundly reshaped contemporary understanding of inherited and complex ocular diseases by revealing the intricate interplay between genomic variation, epigenetic modulation, mitochondrial biology, and environmental stress. Advances in next-generation sequencing have markedly increased diagnostic precision, facilitated genotype–phenotype correlations, and highlighted the substantial proportion of unresolved cases attributable to deep-intronic variants, structural rearrangements, and yet-undiscovered disease genes. At the same time, studies on epigenetic regulation, non-coding RNAs, and mitochondrial dysfunction have emphasized that ocular diseases arise from multilayered biological networks rather than single genetic determinants [142].

Therapeutic development is evolving along similar multidimensional lines. Gene therapy and genome-editing approaches have demonstrated proof-of-concept efficacy and, in selected conditions such as RPE65-associated disease, tangible clinical benefit [27,123]. However, their application remains constrained by biological limitations, immune responses, variable durability, and high implementation costs. Mitochondrial-targeted therapies, RNA-based approaches, and pharmacogenomics illustrate alternative avenues in which molecular insight is beginning to inform personalized therapeutic strategies, although these modalities remain largely at the preclinical or early translational stage.

Looking forward, the integration of genomic data with multi-omics profiling, artificial intelligence, and advanced imaging technologies is expected to refine disease prediction, improve patient stratification, and guide individualized treatment planning. By combining rigorous mechanistic understanding with emerging therapeutic platforms, precision ophthalmology is poised to transition from conceptual framework to practical clinical paradigm. Realizing the full potential of precision ophthalmology will require interdisciplinary collaboration among ophthalmologists, clinical geneticists, bioinformaticians, and AI specialists to harmonize data interpretation, validate predictive models, and translate molecular insights into scalable and equitable care.

## Figures and Tables

**Table 1:** Mechanism-Based Classification of Inherited Retinal and Optic Neuropathies and Current Therapeutic Development.

Mechanistic Class	Representative Diseases (Genes)	Core Cellular Vulnerability	Therapeutic Logic	Major Translational Constraints
Phototransduction/Visual Cycle Dysfunction	LCA (RPE65, GUCY2D) [11,12,13,14,15]; RP (PDE6A/B, RHO) [4]	Impaired chromophore recycling or phototransduction cascade; metabolic stress	Gene augmentation effective when viable photoreceptors remain	Limited window of intervention; reduced benefit in advanced degeneration
Ciliary Trafficking/Structural Integrity Defects	RP (RPGR, USH2A) [4]; Usher syndrome [16,17,18]	Protein mislocalization; outer-segment instability	Early gene replacement; potential RNA-based strategies	Large gene size; delivery constraints; advanced-stage irreversibility
RNA Splicing/Transcript Processing Defects	RP (PRPF genes) [16,17,18]	Aberrant pre-mRNA processing in metabolically demanding photoreceptors	Antisense or splice-modulating therapies	Need for transcript-level validation; tissue accessibility; VUS interpretation challenges
Toxic Metabolite Accumulation	Stargardt disease (ABCA4) [3,19,20,21,22,23]	Lipofuscin accumulation; RPE oxidative stress	Visual-cycle modulation; RNA editing; gene replacement	High allelic heterogeneity; large coding sequence; functional severity stratification required
Mitochondrial Dysfunction (RGC vulnerability)	LHON (MT-ND genes) [11,24,25,26,27]; DOA (OPA1) [28,29,30,31]	Oxidative phosphorylation failure; ATP depletion	Allotopic gene expression; metabolic support; mitochondrial-targeted therapy	Heteroplasmy variability; incomplete penetrance; delivery to RGCs

**Table 2:** Comparison of Genetic Testing Approaches for IRDs and Complex Ocular Diseases.

Testing Method	Strengths/Diagnostic Yield	Limitations	Clinical Indications
Targeted NGS Panels	50–70% yield in many IRDs; high coverage of known genes; low cost; rapid workflow	Misses deep-intronic variants, structural variants, novel genes; limited ability to detect mosaicism	First-line testing for well-characterized IRDs; cost-effective screening in large cohorts
Whole-Exome Sequencing (WES)	Up to 80% yield when combined with phenotype-driven filtering; detects rare coding variants across genome	Limited coverage of non-coding regions; variable exon capture performance; higher rate of VUS	Recommended when panel testing is negative; useful for genetically heterogeneous IRDs
Whole-Genome Sequencing (WGS)	Detects intronic, regulatory, structural, and copy-number variants; highest sensitivity; resolves 10–15% of previously unsolved cases	Higher cost; larger VUS burden; requires advanced bioinformatic interpretation	Complex or unsolved IRD cases; structural variant suspicion; research settings transitioning to clinical use
Mitochondrial DNA Sequencing	Essential for LHON and other mitochondrial optic neuropathies; detects heteroplasmy	Does not detect nuclear mitochondrial gene defects; interpretation of low-level heteroplasmy may be challenging	Optic neuropathies, suspected mitochondrial disorders
Polygenic Risk Scores (PRSs)	Useful for glaucoma, AMD, and pathological myopia risk prediction; integrates multiple common variants	Limited performance in non-European ancestries; not diagnostic; requires population-specific calibration	Risk stratification, personalized screening intervals, and preventive strategies
RNA Sequencing (research use)	Reveals splicing defects and expression abnormalities; valuable when DNA testing is inconclusive	Not widely available clinically; requires tissue accessibility; complex interpretation	Selective use in unresolved IRDs with suspected splicing anomalies

**Table 3:** Approved and Investigational Gene-Based Therapies in Retinal and Optic Nerve Diseases.

Disease/Target	Gene/Pathway	Platform/Delivery	Clinical Status	Main Clinical Signal	Key Translational Challenges
RPE65-associated IRD (LCA/EOSRD)	RPE65	AAV2 (subretinal)	Approved (voretigene neparvovec)	Improved light sensitivity and navigation performance; sustained benefit in many patients	Durability variability; high cost; requires viable photoreceptors; limited indication scope
Choroideremia	CHM	AAV2 (subretinal)	Phase I/II, long-term follow-up	Structural stabilization and variable visual gains	Limited panretinal coverage; inflammation at higher doses; heterogeneous response
X-linked Retinoschisis (XLRS)	RS1	AAV8 (intravitreal)	Phase I/II (halted)	Early anatomical improvement in selected cases	Dose-dependent inflammation; development interruption; uncertain long-term benefit
Achromatopsia (CNGA3/CNGB3)	CNGA3, CNGB3	AAV2/8 (subretinal)	Phase I/II	Modest improvement in contrast sensitivity and photophobia in some cohorts	Developmental cone deficits limit recovery; variable efficacy; ceiling effect
CEP290-associated LCA10	CEP290	CRISPR-Cas9 (EDIT-101)	Phase I/II	Demonstrated feasibility of in vivo genome editing; limited functional improvement in subsets	Editing efficiency; durability unknown; long-term safety; delivery constraints
Usher Syndrome Type 1B	MYO7A	Dual-AAV (subretinal)	Phase I/II	Acceptable short-term safety; early functional trends	Large gene size; recombination efficiency; limited retinal coverage
Optogenetic therapy (late-stage IRD)	Channelrhodopsin/engineered opsins	AAV + external stimulation device	Phase I/II	Restoration of light perception in advanced disease	Low spatial resolution; dependence on stimulation device; adaptation challenges
Anti-VEGF gene therapy (nAMD, DME)	VEGF inhibition	AAV (intravitreal)	Phase II	Reduced injection burden; sustained intraocular expression	Risk of over-inhibition; inflammation; limited controllability of long-term expression
LHON (ND4 allotopic expression)	MT-ND4	AAV2 (intravitreal)	Phase III/extensions	Bilateral visual improvement despite unilateral injection	Variable response; mitochondrial import efficiency; unclear inter-eye diffusion mechanism

**Table 4:** Pharmacogenomic Associations in Ophthalmology and Their Clinical Actionability.

Drug/Therapy Context	Gene(s)	Association/Proposed Mechanism	Evidence Level	Clinical Actionability	Supported by Clinical Guidelines (Yes/No)
Anti-VEGF therapy (nAMD, DME)	VEGFA, KDR	Polymorphisms may influence VEGF ligand–receptor interaction and variability in anatomical/functional response	Moderate; meta-analyses with inconsistent replication	Not used for treatment selection; insufficient predictive power	No
Anti-VEGF therapy (nAMD)	IL-8, CFH, ARMS2/HTRA1	Modulation of inflammatory and complement pathways influencing treatment response	Heterogeneous; population-dependent effects	Experimental relevance only	No
Faricimab (dual VEGF-A/Ang-2 inhibition)	ANG2 pathway regulators	Genetic variation may affect dual-pathway inhibition efficacy	Emerging; limited studies	No current clinical implementation	No
Fingolimod-associated macular edema (FAME)	SPHK1, PLPP3, CYP4F2	Variants alter sphingolipid metabolism and vascular permeability, modifying edema susceptibility	Moderate; supported by functional rationale	Potential monitoring relevance; not standard screening	No
Steroid-induced ocular hypertension	MYOC, FKBP5	Genetic variants influence trabecular meshwork sensitivity and glucocorticoid receptor signaling	Mechanistic and clinical data available; not universally replicated	May justify increased caution; not formally adopted	No
Hydroxychloroquine retinopathy risk	ABCA4, RDH8	Variants may predispose to increased photoreceptor susceptibility to retinoid toxicity	Limited; not consistently replicated	Not used in clinical risk stratification	No
LHON therapy response (idebenone)	mtDNA haplogroups	Haplogroups influence disease penetrance and possibly therapeutic variability	Strong for penetrance; weak for treatment prediction	Used for prognostic counseling, not treatment decision	No

## Data Availability

No new data were created or analyzed in this study. Data sharing is not applicable to this article.

## Abbreviations

The following abbreviations are used in this manuscript:

AAV	Adeno-Associated Virus
ABCA4	ATP-Binding Cassette Subfamily A Member 4
AI	Artificial Intelligence
AMD	Age-Related Macular Degeneration
ANG2	Angiopoietin-2
CFH	Complement Factor H
CNV	Choroidal Neovascularization
CRISPR	Clustered Regularly Interspaced Short Palindromic Repeats
DOA	Dominant Optic Atrophy
DNMT	DNA Methyltransferase
DR	Diabetic Retinopathy
EOSRD	Early-Onset Severe Retinal Dystrophy
GWAS	Genome-Wide Association Study
HDAC	Histone Deacetylase
IOP	Intraocular Pressure
IRD	Inherited Retinal Disease
LCA	Leber Congenital Amaurosis
LHON	Leber’s Hereditary Optic Neuropathy
lncRNA	Long Non-Coding RNA
miRNA	MicroRNA
mtDNA	Mitochondrial DNA
ncRNA	Non-Coding RNA
NGS	Next-Generation Sequencing
OCT	Optical Coherence Tomography
OPA1	Optic Atrophy 1
PM	Pathological Myopia
POAG	Primary Open-Angle Glaucoma
PRS	Polygenic Risk Score
RGC	Retinal Ganglion Cell
RHO	Rhodopsin
RNA-seq	RNA Sequencing
RP	Retinitis Pigmentosa
RPE	Retinal Pigment Epithelium
STGD1	Stargardt Disease Type 1
VEGF	Vascular Endothelial Growth Factor
WES	Whole-Exome Sequencing
WGS	Whole-Genome Sequencing
